# The repurposing of Tebipenem pivoxil as alternative therapy for severe gastrointestinal infections caused by extensively drug-resistant *Shigella* spp

**DOI:** 10.7554/eLife.69798

**Published:** 2022-03-15

**Authors:** Elena Fernández Álvaro, Phat Voong Vinh, Cristina de Cozar, David R Willé, Beatriz Urones, Alvaro Cortés, Alan Price, Nhu Tran Do Hoang, Tuyen Ha Thanh, Molly McCloskey, Shareef Shaheen, Denise Dayao, Amanda Martinot, Jaime de Mercado, Pablo Castañeda, Adolfo García-Perez, Benson Singa, Patricia Pavlinac, Judd Walson, Maria Santos Martínez-Martínez, Samuel LM Arnold, Saul Tzipori, Lluis Ballell Pages, Stephen Baker

**Affiliations:** 1 GSK Global Health, Tres Cantos Madrid Spain; 2 The Hospital for Tropical Diseases, Wellcome Trust Major Overseas Programme, Oxford University Clinical Research Unit Ho Chi Minh City Viet Nam; 3 GSK R&D Stevenage United Kingdom; 4 Division of Allergy and Infectious Disease, Center for Emerging and Re-emerging Infectious Diseases University of Washington School of Medicine Seattle United States; 5 Department of Infectious Disease and Global Health, Tufts University Cummings School of Veterinary Medicine North Grafton United States; 6 Kenya Medical Research Institute Nairobi Kenya; 7 Department of Global Health, University of Washington Seattle United States; 8 University of Cambridge School of Clinical Medicine, Cambridge Biomedical Campus Cambridge United Kingdom; 9 Department of Medicine, University of Cambridge School of Clinical Medicine, Cambridge Biomedical Campus Cambridge United Kingdom; CorpoGen Colombia; Harvard T.H. Chan School of Public Health United States

**Keywords:** Shigella spp, drug discovery, multi-drug resistance, dysentery, diarrhoea, carbapenems, Other

## Abstract

**Background:**

Diarrhoea remains one of the leading causes of childhood mortality globally. Recent epidemiological studies conducted in low-middle income countries (LMICs) identified *Shigella* spp. as the first and second most predominant agent of dysentery and moderate diarrhoea, respectively. Antimicrobial therapy is often necessary for *Shigella* infections; however, we are reaching a crisis point with efficacious antimicrobials. The rapid emergence of resistance against existing antimicrobials in *Shigella* spp. poses a serious global health problem.

**Methods:**

Aiming to identify alternative antimicrobial chemicals with activity against antimicrobial resistant *Shigella*, we initiated a collaborative academia-industry drug discovery project, applying high-throughput phenotypic screening across broad chemical diversity and followed a lead compound through in vitro and in vivo characterisation.

**Results:**

We identified several known antimicrobial compound classes with antibacterial activity against *Shigella*. These compounds included the oral carbapenem Tebipenem, which was found to be highly potent against broadly susceptible *Shigella* and contemporary MDR variants for which we perform detailed pre-clinical testing. Additional in vitro screening demonstrated that Tebipenem had activity against a wide range of other non-*Shigella* enteric bacteria. Cognisant of the risk for the development of resistance against monotherapy, we identified synergistic behaviour of two different drug combinations incorporating Tebipenem. We found the orally bioavailable prodrug (Tebipenem pivoxil) had ideal pharmacokinetic properties for treating enteric pathogens and was effective in clearing the gut of infecting organisms when administered to *Shigella*-infected mice and gnotobiotic piglets.

**Conclusions:**

Our data highlight the emerging antimicrobial resistance crisis and shows that Tebipenem pivoxil (licenced for paediatric respiratory tract infections in Japan) should be accelerated into human trials and could be repurposed as an effective treatment for severe diarrhoea caused by MDR *Shigella* and other enteric pathogens in LMICs.

**Funding:**

Tres Cantos Open Lab Foundation (projects TC239 and TC246), the Bill and Melinda Gates Foundation (grant OPP1172483) and Wellcome (215515/Z/19/Z).

## Introduction

Humanity is currently in the midst of a global antimicrobial resistance (AMR) crisis (Theuretzbacher, 2017; Antimicrobial Resistance Collaborators, 2022). Many of the antimicrobials that we rely on to treat and/or control infections caused by common bacterial pathogens have lost or are rapidly losing their efficacy. This situation has become typical for a host of diseases and pathogens but is arguably epitomised by enteric (diarrhoeal) infections that arise in low and middle-income countries (LMICs). Annually, diarrhoea accounts for approximately 1.3 million deaths globally, with the preponderance of these occurring in young children residing in LMICs (Kotloff, 2017). Due to a lack of other scalable approaches, antimicrobials are a key tool in limiting the impact of these infections, but the trajectory of AMR describes an escalating problem, especially in vulnerable populations.

Diarrhoeal diseases are notoriously complex, as a wide range of pathogens can trigger the syndrome; but of all potential aetiological agents, bacteria belonging to genus *Shigella* pose the greatest AMR threat. Recent estimates suggest that *Shigella* are associated with ~125 million diarrhoeal episodes and 200,000 deaths annually (Khalil et al., 2018). Additionally, *Shigella* along with enterotoxigenic *Escherichia coli* (ETEC), were the most predominant bacterial pathogens in the stools of paediatric diarrhoeal patients in South Asia and sub-Saharan Africa (Kotloff et al., 2013). This study also found that *Shigella* were the most prevalent pathogen in diarrhoeal children aged 2–5 years; a reanalysis of stool samples from this study using more sensitive molecular approaches suggested that burden of *Shigella* may actual be double that of previous estimates (Liu et al., 2016).

Clinical evidence strongly supports the use of antimicrobials to treat severe *Shigella* associated diarrhoea (Kotloff et al., 2018). Appropriate antimicrobial therapy reduces the duration of fever and diarrhoea associated with *Shigella* by 1–2 days, which in turn limits the severity of symptoms and the risk of further complications (Vinh et al., 2000; Christopher et al., 2010). Additionally, the shedding of the pathogen in the stools is restricted by antimicrobials, which then reduce onward transmission. As there is currently no licenced vaccine for *Shigella,* antimicrobials are one of the only reliable mechanisms available for disease control (Levine et al., 2007). However, *Shigella* are highly adept at acquiring multi-drug resistance (MDR) plasmids from other member of the Enterobacteriaceae and resistance mutations are commonly associated with ‘successful’ lineages (Baker et al., 2015; Chung The et al., 2019). Consequently, there has been an ‘arms race’ between the pathogen and antimicrobials, with the WHO ultimately recommending ciprofloxacin, azithromycin, pivmecillinam, and ceftriaxone as the empirical drug choices for *Shigella* infections (Williams and Berkley, 2018). Inevitably, resistance to these antimicrobials has emerged and increasingly resistant organisms, such as extensively drug resistant (XDR) *Shigella sonnei* in Vietnam (Thanh Duy et al., 2020), are spreading across LMICs (Chung The et al., 2019), in travellers returning from LMICs (Baker et al., 2016), and in men-who-have-sex-with-men (MSM) (Baker et al., 2015). Accordingly, the US-CDC has declared AMR *Shigella* a serious global health threat requiring new interventions and the WHO has placed them on the global priority list of antibiotic-resistant bacteria to guide research, discovery, and development of new antibiotics (The World Health Organisation, 2007).

After the genomics revolution of the 1990s, the research community embarked on a series of target-based drug discovery programmes to identify novel antimicrobial compounds. This strategy, because of lack of translation from target-activity to whole-cell activity, produced largely disappointing results (Gwynn et al., 2010). Conversely, phenotypic antimicrobial screens have proven to yield a higher hit rate than target-based approaches with several successful examples in infectious diseases (Gamo et al., 2010; Ballell et al., 2013). Aiming to identify compounds with activity against MDR *Shigella*, we established an academic-industrial collaboration (Ballell et al., 2016), creating a platform to perform in vitro phenotypic screening of ~1.7 million compounds against antimicrobial susceptible and MDR *Shigella*. We interrogated a collection of compounds spanning broad chemical diversity, comprised of a combination of naive chemistry, compounds originating from previous antibacterial drug discovery programs, and currently marketed antimicrobials. Using this approach, we identified several lead chemical classes with previously reported antimicrobial activity, opting to further characterise Tebipenem (Yao et al., 2016), an oral carbapenem, that could be rapidly accelerated into clinical testing to treat infections caused by MDR *Shigella* and other gastrointestinal pathogens.

## Materials and methods

### High-throughput screening of compound collections

We developed a high-throughput screening (HTS) method to identify compounds that could inhibit the growth of *Shigella flexneri* 2457T in Müller Hinton medium (Sigma Aldrich, UK). We adapted a resazurin reduction protocol from Franzblau et al. as surrogate readout for bacterial viability because it provided a stable signal, a low interference level, and was cost effective (Franzblau et al., 1998). Prior to the day of experimentation, one colony was used to inoculate 50 mL Mueller Hinton medium, and the culture was incubated overnight at 37 °C. The next day, 500 μL of the bacterial culture were inoculated into 50 mL fresh Mueller Hinton medium and bacterial growth was measured periodically at 600 nm. When the culture reached OD = 0.4–0.6, the inoculum was diluted to 1 × 10^6^ CFU/mL; this bacterial suspension was added to compound pre-dispensed plates for screening.

The assay was performed in 1536-well polystyrene assay plates (HiBase, μClear, Greiner Bio‐One) and used to screen ~1.7 million compounds from (a) GSK naïve chemical diversity, (b) GSK antibacterial programs, and (c) marketed antimicrobial agents. All commercially available chemicals were purchased from either Sigma Aldrich, UK, AK Scientific, or Fischer Chemicals. The GSK compound screening set was sub-selected from the 8 million compound GSK collection using the principles described by Harper and co-workers (Harper et al., 2004). The process to generate the structural filters to remove compounds bearing undesirable/reactive chemotypes and/or non-developable physicochemical properties has been described previously by Chakravorty et al., 2018. The set of 4000 compounds with known antibacterial pedigree from the GSK antibacterial programs was selected from GSK compound collection using an MIC <1 µM against *E. coli* as cut-off value for selection. The set of marketed antibacterial agents included most commercial antimicrobial agents.

For screening, plates were prepared by dispensing 50 nl of compound from master plates at 1 mM in each well using a LabCyte Echo system. The final assay volume was 5 μl and the final compound concentration was 10 µM. The 11th and 12th columns were growth controls containing 50 nL of DMSO. The 34th and 35th columns contained 50 nL of Moxifloxacin at 20 μM as negative growth controls. Five µl of bacterial inoculum was dispensed into compound pre-dispensed plates using a Multidrop Combi dispenser (Thermo Scientific). Plates were agitated for 10 s to ensure mixing and incubated at 37 °C overnight (16–17 hr). Following overnight culture, 2 µl resazurin solution (Resazurin tablets for milk testing, BDH) was added to each well and incubated at room temperature in the dark for 3 hr. Fluorescence intensity was measured using an Envision plate reader (PerkinElmer). For IC50 and IC90 determination, data were normalised by using DMSO as positive control (100% bacterial growth) and 20 μM moxifloxacin as negative control (0% bacterial growth). Data were analysed using Base software and statistical cut-off analysis was used for ‘hit’ selection following a standard GSK protocol. To calculate the statistical cut-off, percentage growth inhibition was calculated per well according to the plate controls, these values were aggregated to calculate the robust mean and standard deviation (SD) of the distribution. The robust mean was calculated starting from the median and the MAD of the distribution with an iterative process that aimed to remove outliers. Once the mean and SD were calculated, all compounds above mean + 3 SD were selected as primary hits.

After the initial screen, we generated fresh samples of the primary compound hits and tested them in duplicate against *S. flexneri* 2457T at 10 μM to confirm activity. The confirmed hit list was narrowed by applying the following filters: a 30% cut-off value, absence of undesirable structural features (e.g. electrophiles, peroxides, and Michael acceptors) and reduced physicochemical risk (maximum five aromatic rings and PFI <8)(Leeson and Young, 2015). The resulting compounds were progressed to dose-response assays, performed in duplicate at a concentration of 100–0.00169 μM against *S. flexneri* 2457T. Compounds with an IC50 <1 μM were clustered and progressed to evaluation against two MDR clinical *Shigella* isolates from Vietnam (*S. flexneri* EG478 and *S. sonnei* 02–1182). This additional evaluation was performed in duplicate, with compounds displaying an inhibition halo at 10 μM in both isolates progressed to a dose-response evaluation at compound concentrations 10–0.078 μM. Compounds that exhibited an MIC <10 μM in both isolates were selected.

Three GSK control compound sets were used to validate our HTS approach (Coma et al., 2009). Firstly, a 10,000-compound validation set was used to fine tune Z´and S/B ratio values in HTS conditions. The 0.65% hit rate obtained in this test was aligned with the rate expected for whole cell screenings. Secondly, we used a 1000-compound nuisance set containing compounds selected for their high Inhibition frequency index (IFI; defined as the % of HTS campaigns in which a compound has shown >50% inhibition at 10 μM) to identify assays with high susceptibility to nuisance mechanisms, which can lead to identification of a high rate of false positive compounds. We obtained a hit rate of 0.39 with this set, corresponding to a low false positive rate. Lastly, an antibacterial tool compound set contained an ad hoc set of marketed antimicrobials with different modes of action. This set was evaluated using the two prioritised readout systems (resazurin and XTT) and was used to mitigate the risk of biasing the hit identification by the mechanism of action as both readouts displayed similar IC_50_ values in all compound families.

### Antimicrobial susceptibility testing and time kill curves performed in clinical isolates

Selected compounds, including Tebipenem, were profiled against a panel of 338 clinical isolates of enteric pathogens comprised of *Shigella* (Vietnam and Kenya; n = 135), non-typhoidal *Salmonella* (Vietnam; n = 117), *Campylobacter* (Vietnam; n = 75), and various pathogenic *E. coli* species (Vietnam; n = 7) (Butcher et al., 2014). The antimicrobial susceptibility profile of these organisms is described in Supplementary file 1.

Compound screening and dose response assays have been described previously (Van Voorhis et al., 2016; Wiegand et al., 2008). Briefly, the selected bacteria were cultured on the Luria-Bertani agar overnight at 37 °C (*Campylobacter* isolates were grown in 2.5% Laked Horse Blood cation-adjusted Mueller Hinton medium (CAMHB, Sigma Aldrich) and incubated in microaerophilic conditions (10% CO_2_, 5% O_2_ and 85% N_2_) at 42 °C); colonies were added to sterile PBS to form 0.5 McFarland solutions. All chemical testing was performed at an initial concentration of 10 μM. Bacterial suspensions were evenly spread on Mueller-Hinton agar by sterile cotton swabs (Medical Wire – MWE). To test the chemicals, 5 μl of 10 μM solutions were pipetted onto the surface of the media and 5 μl of 10 μM of ciprofloxacin/meropenem was used as a positive control and sterile PBS as a negative control. Plates were incubated overnight at 37^o^ C. The activity of the chemical was recorded as being active (yes) or non-active (no) for each of the tested chemicals. A non-active result was recorded when there was no evidence of bacterial inhibition, and the culture area was indistinguishable from the negative control. An active result was recorded when there was an obvious zone of inhibition of bacterial growth and the observed zone was comparable to that of the positive control, that is, an area without bacterial growth where the media could be observed.

MICs were evaluated using the micro dilution assay in Müller Hinton broth using 1 mM chemical stock solutions, and a final concentration of 5 × 10^5^ CFU/mL bacteria. Twofold serial dilutions were prepared in 96-well plates at concentration ranging from 1 μM to 7.8 nM. 100 μL bacteria were added and incubated at 37^o^ C overnight; 10 μl of each solution was dropped onto Luria-Bertani agar and incubated overnight at 37 °C for the detection of bacterial growth. Results were interpreted as minimal concentration necessary to inhibit growth.

Time-kill curve assays were performed in 20 mL volume in 50 mL tubes by culturing 5 × 10^5^ CFU/mL *Shigella* spp. in Müller Hinton broth in the presence of four antimicrobial concentrations in doubling dilutions ranging from 0.5xMIC to 4xMIC. Bacteria were grown with agitation at 200 rpm at 37 °C and monitored over a time-course of 24 hr (0, 2, 4, 6, 8, and 24 hr). For each concentration and time point, 100 μL of bacterial culture were diluted and used to inoculate Luria-Bertani agar plates that were incubated at 37 °C overnight. Next, bacterial colonies were enumerated.

### Spontaneous resistant mutants

The frequency of spontaneous resistant mutants (FoR) was determined for Tebipenem, Moxifloxacin, ciprofloxacin, ampicillin, mecillinam, ceftriaxone, and rifampicin. Bacterial inoculum without drug was used as positive control. The bacteria inoculum (1 × 10^5^ CFU/mL) for the assay was prepared by diluting a log phase incubated at 37 °C for 3–4 hr. Mueller Hinton agar plates were prepared containing 4xMIC and 10xMIC and inoculated with different concentrations of bacteria (1 × 10^9^, 1 × 10^8^, and 1 × 10^7^ CFU/mL). Plates were prepared in duplicate and incubated overnight at 37 °C. The frequency of spontaneous resistant mutants was calculated by using the formula: FoR = resistant CFUs/inoculum CFUs. The mean of the two plates was reported.

### Synergistic assays

Before initiating drug combination assays, the MIC values for both compounds were determined against *Shigella* (Sf2457T), *Salmonella* (St14028)*,* and *E. coli* (EcDH5a) isolates. Standard checkerboard titration method using isobolograms was used to study the in vitro interaction between Tebipenem and azithromycin, ciprofloxacin, ceftriaxone, mecillinam and an LpxC inhibitor. 20 μM moxifloxacin was used as negative control (100% bacterial growth inhibition) in one column and DMSO as positive control (100% bacterial growth). A total of 25 µl of 1 × 10^6^ CFU/mL bacterial culture was added to each well of a 384-well plate (μClear; Greiner Bio‐One). Plates were incubated overnight at 37 °C. Next, 10 µl of resazurin were added and plates were incubated at room temperature for 3 hr. Bacterial growth inhibition was measured by fluorescence in a Envision plate reader (PerkinElmer). All assays were performed in duplicate. Combination studies with clinical isolates were performed in 96-well plates (Greiner) adding 200 μl of 1 × 10^6^ CFU/mL bacterial culture and following the same checkboard titration method as described above. The range of final working concentrations of each antimicrobial was adjusted based on the MIC of each selected organism.

MICs were determined for drug A and drug B alone and in combination. The MIC of both drugs in combination were expressed as fractions of the MIC of the drug alone normalised to 1, which represented the Fractional Inhibitory Concentration (FIC). The sum of FIC was expressed using the following equation: FIC = (MIC of drug A in combination / MIC of drug A alone) + (MIC of drug B in combination / MIC of drug B alone) (Le Minh et al., 2015; Petersen et al., 2006; Hall et al., 1983; European Committee for Antimicrobial Susceptibility Testing (EUCAST) of the European Society of Clinical Microbiology and Infectious Dieases (ESCMID), 2000).

### Extrapolation of orapenem doses approved in paediatric population to other animal species

Pharmacokinetic parameters of Orapenem in paediatric population at the approved doses (4–6 mg/kg, bid) were obtained from Sato et al. and pharmacokinetic parameters in rats were generated in house (Sato et al., 2008). Information regarding Orapenem oral bioavailability was extracted from Kijima et al. (Sato et al., 2008). For the calculation of Tebipenem pharmacokinetic behaviour in different animal species (i.e. mouse and piglet) conserved intrinsic clearance (CL_int_) and distribution volume (Vss) across the different animal species were assumed. A mono-compartmental pharmacokinetic (PK) model was used for PK simulation using matlab simbiology software (Hosseini et al., 2018). The equivalent doses in the different animal species were calculated using Tebipenem free drug t/MIC as comparable parameter.

### In vivo pharmacokinetic analysis in mice

All mice experiments were performed in female C57 mice obtained from Charles River Laboratories, (Wilmington, MA) and housed in cages in groups of three animals with water and food ad libitum. Animals were acclimated for 7 days prior to infection. At the beginning of the experiment the mice weighed between 20 and 30 g. Tebipenem was dissolved in 20% Encapsine (Sigma Aldrich), 5% DMSO (Sigma Aldrich) in saline solution (Sigma Aldrich) at 0.5 mg/ml for intravenous administration and in 1% Methylcellulose (Sigma Aldrich) in water at 5 mg/ml for oral administration. Tebipenem pivoxil was dissolved in 1% (w/v) Methylcellulose (Sigma Aldrich) in water at 5 mg/ml for oral administration.

For blood PK analysis, two 15 µl of tail blood were collected per time and per animal by micro-sampling at 0.08, 0.25, 0.5,1, 2, 4, 6, and 24 hr for intravenous pharmacokinetics and 0.25, 0.5, 0.75, 1, 2, 4, 6, and 24 hr for oral pharmacokinetics. Blood samples (15 µl) were mixed and vortexed with 30 µl sterile water and snap frozen at –80 °C prior to analysis. To generate the data, the blood samples were thawed at ambient temperature and 10 µL were mixed with ACN: MeOH (80:20), filtered (0.2 µm filter plate, Whatman) and analysed by LCMS.

An acquity ultra-performance liquid chromatography (UPLC) system (Waters Corp., Milford, MA, USA) coupled to a triple quadrupole mass spectrometer (API 4000, AB Sciex, Foster City, CA, USA) was used for the detection of Tebipenem free base. The chromatography separation was performed at 0.4 mL/min in a Acquity UPLC BEH C18 column (50 × 2.1 mm i.d., 1.7 mm; Waters Corp.) at 40 °C with Acetonitrile (Sigma Aldrich) and 0.1% formic acid as eluents. Calibration curves were included to quantify blood samples, with a lower limit of quantification (LLOQ) of 2.5 ng/mL and a high limit of quantification of 50,000 ng/mL. Along the analytical sequence, several quality control (QC) samples were included (low, medium, and high concentrations); in all cases the deviation of QC was lower than 20%. Samples under LLOQ were excluded from the pharmacokinetic analysis. Pharmacokinetics parameters were calculated using Phoenix WinNonlin (Certara NY, US).

### In vivo pharmacokinetic analysis in piglets

Blood samples for analysis of orally administered Tebipenem pivoxil pharmacokinetics in gnotobiotic piglets were taken from the same animals involved in the therapeutic efficacy study described below. Tebipenem pivoxil was dissolved in 1% (w/v) Methylcellulose (Sigma Aldrich) in water at 5 mg/ml for oral administration. Four whole blood samples and were taken from the Tebipenem pivoxil treated gnotobiotic piglets. Blood was collected from the jugular veins while manually restraining the piglets. Blood was collected at 0.5, 1, 2, and 4 hr after administration of the first dose of Tebipenem pivoxil. Blood samples were centrifuged at 2,200 x g for 10 min at 4 °C. Plasma was extracted and stored at –80 °C. Pharmacokinetic analysis was performed as above described.

### Orapenem intestinal distribution studies

To determine the distribution of Tebipenem pivoxil and free base in the different parts of the intestine, Tebipenem pivoxil was orally administrated at 50 mg/kg to four C57 mice (5 mg/ml in 1% (w/v) Methylcellulose). One animal was euthanised at 0.25, 0.5, 1, and 4 hr respectively and 2.5–3 cm sections of the intestine (duodenum, jejunum, ileum, caecum, and colon) were collected and frozen at –80 °C. For quantitative LCMS analysis of intestine homogenate, samples were thawed at ambient temperature, mixed with water (1:1) and homogenised by turrax (10 sec). Subsequently, 20 µL of homogenate were mixed with 200 µl of ACN: MeOH (80:20) and filtered using 0.2 µm filter plate (Whatman). LCMS analysis was performed as described in previous section. For MALDI imaging, 50 mm of the central part of each intestinal section were cut and mounted in a sample disc on dry ice with a solution of 1% methylcellulose (Sigma Aldrich, UK). Next, tissue sections of 12 µm (sagital sections) were obtained using a cryostat (Leica CM3600; Leica Microsystems Inc, Wetzlar, Germany) at –20 °C and collected in conductive-coated slides for MALDI Imaging (Bruker Daltonic). After the ‘MALDI’ section was obtained a consecutive section for histology was taken. Prior to matrix coating, samples for MALDI were optical scanned in a PathScanner. The matrix deposition was conducted in a sprayer ImagePrep System (Bruker Daltonic) which uses vibrational vaporisation technology. As matrix solution *α*-CHCA (α-Cyano-4-hydroxycinnamic acid (Sigma Aldrich) dissolved in 70:30 methanol/water (v/v) containing 0.2% TFA at 7 mg/mL was used. MALDI MSI data were obtained using a Bruker UltrafleXtream MALDI TOF/TOF (Bruker Daltonic, Germany)).

Standard solution of Tebipenem and Tebipenem Pivoxil at 0.1 mg/ml were spiked in control mice intestines to optimise the analysis parameters. Both compounds were analysed in positive mode using a LIFT method. For Tebipenem, parent compound was 384.32 and the detection mass window between m/z 0–400. 298.164 were the most intensive fragment. For Tebipenem pivoxil, the MS/MS transition was 497.5/383.3. We used a laser shut at 85% power with a 50 μm step size, 100 times at raster spot in random wak mode. FlexImaging 4.1(bruker Daltoninc, Germany) were used for the data visualisation. MS/MS transition for Tebipenem was 384.1/298.1 and for Tebipenem pivoxil 497.542/298.1 All ions were displayed with a mass tolerance of ±0.2 Da. A TIC normalisation (divide all spectra by the mean of all data point) was applied.

MALDI Imaging is not a quantitative technique, therefore, the colour scale of images is relative to the maximum intensity (100 corresponds with the maximal signa in each slide). For histology analysis, consecutive MALDI sections were collected and stained with Hematoxilin-Eosin staining (Sigma Aldrich) following manufacturer’s protocol and scanned in a Path Scanner IV.

### In vivo mouse efficacy studies

The in vivo efficacy of Tebipenem pivoxil, Tebipenem free-base and Ciprofloxacin was evaluated using a previously published mouse model of shigellosis using *S. flexneri* lux1 (McCloskey et al., 2019). *S. flexneri* lux1 expresses a bacterial luciferase reporter as previously described and was created from *S. flexneri* M90T Sm (ATCC BAA-2402) purchased from the American Type Culture Collection (ATCC, Manassas, VA). Broth cultures of *S. flexneri* lux1 were grown at 37 °C in Trypticase soy broth (TSB) with agitation at 220 rpm.

For infections, an overnight *S. flexneri* lux1 broth culture containing 30 µg/mL streptomycin (selection of lux1) and 0.1% sodium deoxycholate (for expression of *Shigella* invasion proteins [McCloskey et al., 2019]) was diluted 1:100 and grown to log-phase (3–4 hr). Log-phase bacteria (OD_600_ ~ 0.4) were centrifuged, and the resulting pellet was washed in DPBS without calcium and magnesium (Thermo Fisher Scientific). Bacteria were centrifuged again, and pellet was resuspended in DPBS to the target concentration of 5 × 10^7^ *S. flexneri*/200 µL (10 mL/kg). Each dose of bacteria was confirmed with luminescence using an EnVision Plate Reader (Perkin Elmer) for *S. flexneri* lux1. Female B6 mice (Jackson Laboratories) aged 9–10 weeks were weighed and infected IP with 5 × 10^7^ CFU/mouse (total n = 10 per group). Antimicrobials were suspended in oral vehicle (1% carboxymethyl cellulose), and mice were administered antimicrobials or vehicle control PO at 2- and 16 hr post infection (p.i.). Ciprofloxacin was used as positive control and administered at 40 mg/kg of body weight. Mice were euthanised 24 hr post infection and the small intestine and cecum/large intestine were collected. Gastrointestinal tissue was weighed, resuspended in 1 g/mL DPBS, homogenised, and plated on trypticase soy agar plates with 30 µg/mL streptomycin to quantify *S. flexneri* CFUs. Experiments were performed in biological duplicates. Data was analysed independently for the small and large intestines using an analysis of variance after a log transformation, comparing active groups back to control with a Dunnett’s post hoc test. CFUs at the limit of quantification of 80 or below were imputed at 80 units after a stability analysis which showed that taking smaller values did not affect the overall conclusion. The data from the ciprofloxacin treatment, which were all <80 CFU was not analysed.

### In vivo gnotobiotic piglet studies

The primary parameter of drug efficacy was determined by a decrease in symptoms in drug-treated animals compared with infection-control animals and ciprofloxacin-treated animals. Additional parameters of efficacy were the evolution of bacterial counts in faeces and histopathology results (quantification of bacterial intestine colonisation and mucosal lesions at the end of the experiment; day 6 after infection). For infection, *S. flexneri* 2457T was grown overnight on blood agar plate from which a single colony was further grown in tryptic soy broth overnight. Bacterial OD_600_ was measured and adjusted to 10^9^ CFU/ml. The bacterial suspension was washed, centrifuged, and resuspended in sterile PBS. The bacterial concentration was confirmed by OD and plating dilutions on agar plates. Limit of detection was 100 CFU/g.

Animals were derived via caesarean section, maintained in sterile microbiological isolators, and fed three times daily with human infant milk replacer. Twelve piglets aged 24 hr were orally inoculated with 1 × 10^9^ CFU/ml *S*. *flexneri*. Eighteen hours after the infection, gnotobiotic piglets received oral administration of Tebipenem pivoxil, ciprofloxacin (control for effective treatment), or no treatment (infection control). Four gnotobiotic piglets were infected and treated with Tebipenem pivoxil at 50 mg/kg, two piglets were infected and received ciprofloxacin at 30 mg/kg, two piglets were not infected and received Tebipenem pivoxil treatment (control for drug effect) and four piglets were infected and received formulation vehicle (1% methyl cellulose, control for infection). Treatments were given twice a day for 5 days. Clinical manifestations such as diarrhoea, depression, loss of appetite and dehydration were monitored and recorded daily. Diarrhoea was determined based on faecal consistency and recorded three times daily: 0 no diarrhoea, 1-soft/pasty, 2-semi-liquid/mucoid, 3-watery and 4, water yellow white. Body weight was taken daily except on day 1 post infection. Faecal samples were collected from the animals once on day 0 and twice on days 1–5 post infection. Piglets were euthanised on day 6 after infection. At euthanasia, animals were heavily sedated by intramuscular injection of ketamine/xylazine (100 mg/5 mg) followed by intracardiac administration of SomnaSol (100 mg/kg). Necropsy was performed 2 hr after the last drug dose. At necropsy, gastrointestinal tissues were harvested for histological examination and quantification of intracellular bacterial load. Gut content was also collected for bacterial burden enumeration. For the determination of bacterial burden in faeces, 100 µl or 100 µg of faeces was mixed with 900 µL PBS. For gut sections (caecum and colon), the tissue was weighted, placed in a sterile 15 ml conical tube, and washed three times with sterile PBS. A 1:9 ratio of tissue and sterile PBS was transferred to GentleMACS M tube then homogenised using the GentleMACS machine. The suspension was serially diluted to 1/10, and 20 µL of each dilution were plated in triplicate and incubated overnight at 37 °C. The following equation was used to calculate CFU/ml from the original aliquot / sample: CFU per ml = Average number of colonies for a dilution x 50 x dilution factor.

### Histopathology of guts sections from infected gnotobiotic piglets

Sections of the large intestine were taken from tebipenem treated and untreated animals and fixed in paraffin blocks. Sections were sliced in a microtome and subjected to deparafinisation and rehydration by 10 min in Xylene (x2), 5 min in 100% ethanol, 3 min in 95% ethanol, 3 min in 80% ethanol, 3 min in 70% ethanol, 3 min in deionised water. Sections were blocked for 5 min in hydrogen peroxide followed by 5 min in deionised water. Tissue slides were steamed for 20 min of 200 ml of 1 x iCitra solution, before cooling to ambient temperature for 20–30 min prior to three washes for 5 min in deionised water and 5 min in TBS. Sections were blocked with 1 x Blocking solution in deionised water (Biogenex, HK085) and incubated at ambient temperature for 8 min before rinsing in 1 x TBST. Sections were incubated with 100 μl of primary antibody mix (Rabbit anti-*Shigella* at 1:250 dilution in TBS) and incubated in humid chamber at ambient temperature for 1.5 hr prior to draining and washing twice for 5 min in TBST. Sections were blocked with 3% hydrogen peroxide for 5 min and washed twice for 5 min in TBST before the addition of 100 μl of secondary antibody (Goat-anti-rabbit HRP) at 1:1000 dilution. Sections were incubated in a humid chamber at ambient temperature for 40 min. Sections were drained and washed twice for 5 min in TBST and once for 5 min in TBS. For counterstaining the sections were incubated in hematoxylin at ambient temperature for 5 min, rinsed in tap water for 3 min, before dipping in bluing solution (0.1% Sodium Bicarbonate) for 1 min, and rinsing deionised water for 3 min. Tissue was dehydrated twice for 3 min in 95% ethanol, twice for 3 min in 100% ethanol, and twice dipped in Xylene. A few drops of Ecomount mounting media were added to the section prior to the addition of a coverslip. Sections were left to dry overnight prior to imaging and photographs on a light microscope.

## Results

### High-throughput screening of compound collections

**Figure 1.**
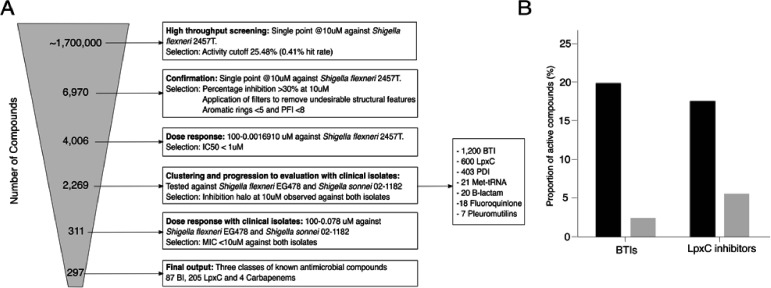
Screening compounds for antibacterial activity against *Shigella* spp. (**A**) Flowchart outlining the screening process in six key stages: (1) identification of compounds with antibacterial activity against *S. flexneri* 2457T from an initial set of ~1.7 million chemicals at a concentration of 10 µM. (2) Confirmation of original hits with activity against *S. flexneri* 2457T at 10 µM with application of additional described filters. (3) Dose response against *S. flexneri* 2457T selecting compounds with IC50 of <1 µM. (4) Testing compounds with antibacterial activity against *S. flexneri* EG478 and *S. sonnei* 02–1181 (clinical isolates from Vietnam) at a concentration of 10 µM (leading to 2,269 compounds in seven classes). (5) Dose response against *S. flexneri* EG478 and *S. sonnei* 02–1181 selecting compounds with IC50 of <1 µM against both organisms. (6) Identification of the final 297 compounds in three classes. (**B**) Bar charts showing the activity profile (proportion of chemicals with antibacterial activity) of BTIs and LpxC inhibitors against the *S. flexneri* 2457T laboratory isolate (black) and both clinical isolates (*S. flexneri* EG478 and *S. sonnei* 02–1181; grey).

We conducted a large antibacterial screening campaign, incorporating both GSK proprietary and non-proprietary compounds, to identify new chemical entities (NCE), repurposing and repositioning opportunities that may provide an alternative for treating infections caused by MDR/XDR *Shigella*. The chemical diversity included the GSK screening collection (~1.7 million compounds of unbiased chemical diversity), 4000 compounds with known antibacterial pedigree (MIC <1 µM against *E. coli*) including chemicals from antibacterial programs at GSK and existing commercially available antimicrobials. A flowchart of the entire screening procedure is shown in Figure 1A.

Compounds were tested in 1,536-well plates at a concentration of 10 µM for their ability to inhibit the growth of *S. flexneri* 2457T in liquid medium using resazurin reduction as a surrogate of bacterial viability. A mean OD with three standard deviations cut-off was used and a robust Z’ mean value of 0.7 and a signal/noise ratio of 12 were obtained from the screening. At an average statistical activity cut-off of 25.48%, 6970 primary hits were identified, representing an overall hit rate of 0.41%, which is lower than the typical hit rates for phenotypic high throughput screens (~1%). In the confirmation step, primary hits were tested in duplicate against *Shigella flexneri* 2457T at a final concentration of 10 μM; compounds inhibiting <30% bacterial growth, displaying undesirable structural features (such as electrophiles, peroxides, Michael acceptors) or low developable physicochemical properties ( < 5 aromatic rings and PFI <8-) were removed (Leeson and Young, 2015). Compounds fulfilling confirmation criteria (n = 4006) were progressed to dose response inhibition curves against *Shigella* 2457T, which were performed in duplicate. Ultimately, 2269 compounds in seven classes (bacterial topoisomerase inhibitors (BTIs), inhibitors of UDP-3-*O*-(*R*-3-hydroxymyristoyl)-*N*-acetylglucosamine deacetylase (LpxCs), microcin PDI, Met-tRNA inhibitors, β-lactams, fluoroquinolones, and pleuromutilins) were identified that inhibited the growth of *Shigella flexneri* 2457T with an IC50 ≤1 µM (Figure 1A).

The 2269 filtered active compounds were progressed to evaluation against two clinical isolates of MDR *Shigella* originating from Vietnam, *S. flexneri* EG478 and *S. sonnei* 02–1181; the antimicrobial susceptibility profiles of these organisms are shown in Supplementary file 1. Compounds were tested in solid medium at 10 µM against both isolates. From the 2269 compounds evaluated, 297 generated an MIC ≤10 µM against both organisms. The 297 compounds belonged to only three already known antibacterial classes: β-lactams, BTIs, and LpxCs. Notably, we did not identify any hits representing new chemical diversity, NCEs, or compounds with novel modes of action. Additionally, the majority of BTIs, LpxCs, and carbapenems had a notable loss of potency when tested against the two clinical MDR *Shigella* isolates in comparison to the original laboratory reference organism (Figure 1B). This trend highlights the requirement for representative contemporary bacterial isolates for drug screening campaigns and the development of novel antimicrobials.

Given the general availability and acceptability of carbapenems globally, efforts to determine their potential to be progressed as treatment against shigellosis were prioritised. Meropenem, Tebipenem, and Ertapenem were identified via the screening and exhibited the greatest bactericidal activity against the two MDR *Shigella* clinical isolates (Supplementary file 2). Consequently, these compounds were prioritised for evaluation. Notably, Tebipenem pivoxil is administered orally, while Meropenem and Ertapenem are administered parenterally or intramuscularly. Therefore, Tebipenem was of specific interest as the pivoxil prodrug has good oral bioavailability and is commercially available in Japan (Jain et al., 2018).

### Tebipenem as broad-spectrum antimicrobial for treating Shigella infections

We considered that an oral drug with potential for accumulation in the gastrointestinal tract would be a suitable profile for treating invasive enteric pathogens; therefore, we reasoned that Tebipenem may be the most appropriate identified compound that could be accelerated into further investigations for treating MDR/XDR *Shigella* infections. Using in vitro assays, we found that Tebipenem inhibited the growth of *S. flexneri* 2457T with a potency comparable to current first line antimicrobials in broadly susceptible organisms, including both ciprofloxacin and ceftriaxone (Table 1).

**Table 1.** MIC values of different antimicrobials against *Shigella flexneri* ATCC 700930.

Antimicrobial	IC_90_ (µg/ml) *
Ciprofloxacin	0.020
Mecillinam	0.058
Azithromycin	7.49
Ceftriaxone	0.067
Tebipenem	0.010

* Data shown are the mean of triplicates.

**Figure 2.**
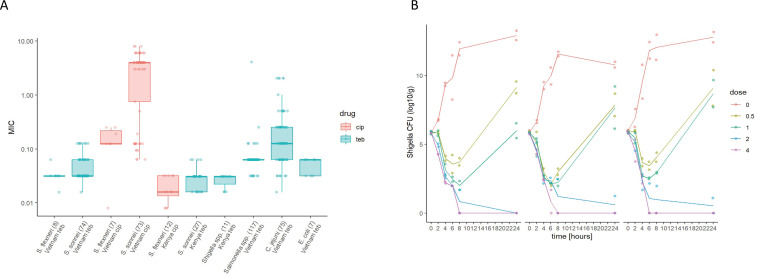
The in vitro activity of Tebipenem against various enteric pathogens. (**A**) Boxplots showing the log of the minimum inhibitory concentrations (MIC; µg/ml) of Tebipenem (teb) (blue) and ciprofloxacin (cip) (red) for clinical isolates of *Shigella* spp. (*sonnei* and *flexneri*) from Vietnam and Kenya (highlighted) and a range of other enteric pathogens. The boxes show the interquartile range of MICs (median line), with individual data points, whiskers show the highest and lowest MICs; the number of isolates screened are highlighted on the x axis. MICs were performed in duplicate; a third replicate was performed if the initial data did not match. Time kill curves of 0 x (red), 0.5 x (light green), 1 x (dark green), 2 x (blue), and 4 x (pink) MIC Tebipenem over 24 hr against (**B**) *S. flexneri* EG478, (**C**) *S. flexneri*_01_0417, and (**D**) *S. sonnei*_02_1181; counts shown in CFU/ml and represent the mean ± the standard deviation of three biological replicates.

Given the propensity of *Shigella* spp. to horizontally acquire AMR genes (Thanh Duy et al., 2020), we additionally assessed the activity of Tebipenem against a collection of 82 contemporary clinical *Shigella* isolates (*sonnei* and *flexneri*) from Vietnam and 53 (multiple species) from Kenya. The compound exhibited consistent in vitro activity against all clinical isolates of *Shigella*, with MIC values ranging from 0.04 to 0.3 µM (Figure 2A). Particularly, Tebipenem generated substantially lower MICs than ciprofloxacin in isolates from Vietnam and had good in vitro activity against all MDR and XDR *S. sonnei* (Figure 2A). Severe diarrhoea can also be caused by a range of other bacterial pathogens; therefore, we additionally performed in vitro MIC testing against a collection of 117 *Salmonella*, 75 *Campylobacter jejuni,* and seven *E. coli* from diarrhoeal cases in Vietnam (Duong et al., 2018). Tebipenem retained good bactericidal activity against all three genera with a median MIC of <0.1 µM against all organisms (Figure 2A).

We additionally evaluated the frequency of spontaneous Tebipenem resistance mutations in vitro using *S. flexneri* 2457T at concentrations of 4 x and 10 x MIC. The frequency of spontaneous resistance against Tebipenem was calculated as <1.2 × 10^–9^, which is low and was comparable to other currently available antimicrobials, including the fluroquinolones (Supplementary file 3).

We next conducted time kill assays of Tebipenem against three clinical isolates of *Shigella* (two *S. flexneri* and one *S. sonnei*) from Vietnam. These assays were performed in triplicate at four increasing concentrations (0.5x to 4x MIC) over 24 hr. The time kill experiments further demonstrated that Tebipenem had protracted antibacterial activity against *Shigella* at higher concentrations (2–4 x MIC). Population rebounds occurred at lower concentrations (0.5–1 x MIC) after 6 hr exposure in all three isolates (Figure 2B, C and D). Taken together, these in vitro data indicate that Tebipenem administered as a prodrug may be a practical alternative for oral treatment of severe *Shigella* associated diarrhoea (and potentially other enteric pathogens) in locations with a high burden of MDR and fluoroquinolone resistant organisms.

### Synergistic combinations of Tebipenem pivoxil

**Figure 3.**
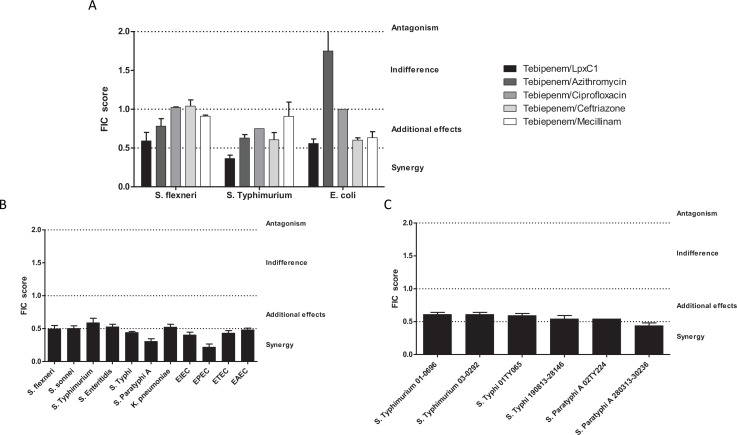
In vitro synergy of Tebipenem with common antimicrobials. (**A**) Bar chart showing the average (assays performed in duplicate) fractional Inhibitory Concentration (FIC) index value determining the synergy/antagonism potential of Tebipenem in combination with PF-5081090 (LpxC inhibitor), azithromycin, ciprofloxacin, ceftriaxone, and mecillinam (highlighted) against laboratory isolates of *S. flexneri*, *S. typhimurium*, and *E. coli* (labelled on x axis). The scale of interaction is highlighted on the right. (**B**) Bar chart showing the average FIC index value to assess the synergy/antagonism potential of Tebipenem in combination with an LpxC inhibitor against a range of clinical isolates of enteric bacteria (labelled on x axis) (assays performed in duplicate). (**C**) Bar chart showing the average FIC index value to assess the synergy/antagonism potential of Tebipenem in combination with azithromycin against a range of clinical isolates of invasive and non-invasive *Salmonella* (labelled on x axis) (assays performed in duplicate).

Given the potential issues associated with widespread usage of an oral carbapenem, we performed preliminary experiments to explore the possibility of synergistic combinations between Tebipenem and the current WHO approved drugs for shigellosis; azithromycin, ciprofloxacin, ceftriaxone, and mecillinam. We additionally progressed one of our previous observations by combining Tebipenem with the commercially available LpxC inhibitor, PF-5081090 (García-Quintanilla et al., 2016). Initially, these five combinations were tested against laboratory isolates of *Shigella*, *Salmonella,* and *E. coli* (Sf2457T, St14028, and EcDH5a, respectively). Tebipenem exhibited partial synergy in combination with the LpxC inhibitor, reducing the MIC against Tebipenem > 10 fold in all three organisms (Figure 3A). Similarly, Tebipenem demonstrated partial synergy with azithromycin against *S. flexneri* and *S*. Typhimurium, but this combination appeared to show indifference or antagonism against *E. coli*. In combination with ciprofloxacin, ceftriaxone, and mecillinam, Tebipenem exhibited indifference or partial synergy against all three organisms (Figure 3A).

We further assessed the potential for synergism between Tebipenem with PF-5081090 and azithromycin against a panel of alternative Gram-negative MDR clinical isolates. The combination of Tebipenem/LpxC inhibitor exhibited indifference or partial synergy in vitro against all tested enteric pathogens (*Salmonella* spp.*, Shigella* spp.*, K. pneumoniae,* and *E. coli*). Notably, the combination of Tebipenem with azithromycin was particularly synergistic in vitro against a range of invasive and non-invasive *Salmonella* isolates, with FIC scores of ~0.5 (Figure 3B).

### Tebipenem efficacy and pharmacokinetic properties in animal models of Shigella infection

Tebipenem pivoxil, under the brand name Orapenem, is currently approved in Japan for the treatment of respiratory tract infections. Aiming to confirm the potential of repurposing Tebipenem pivoxil for the treatment of enteric infections through in vivo experimentation, we progressed the drug for pharmacokinetic and efficacy studies in animal models. The doses for pharmacokinetics and efficacy studies were extrapolated from the approved doses in children (4 and 6 mg/kg bid) and we calculated equivalent doses in terms of systemic exposure in the animal species used for the efficacy and pharmacokinetic studies (described in Materials and methods).

**Figure 4.**
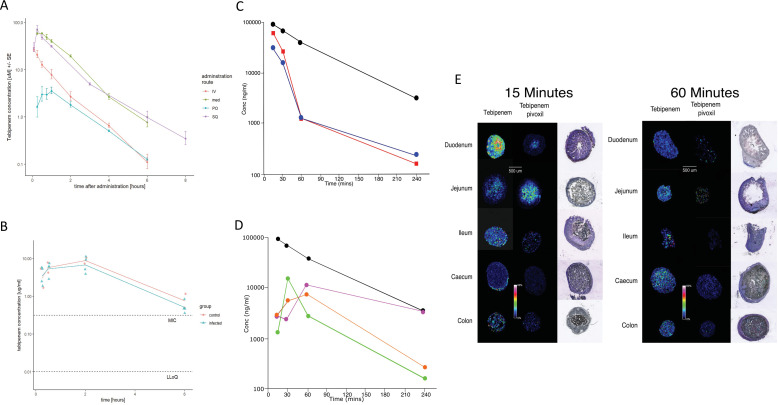
The pharmacodynamics of Tebipenem in blood and the gastrointestinal tract of experimental animals. (**A**) The blood pharmacokinetics of Tebipenem in mice (n = 3 per treatment) over 6 hr after the administration of 5 mg/kg intravenous Tebipenem (red), 39 mg/kg subcutaneous Tebipenem (pink), 39 mg/kg oral Tebipenem, and 50 mg/kg oral Tebipenem pivoxil. Data points represent mean ± the standard deviation. Notably, the drug concentration in all preparations remained above the estimated MIC of *S. flexneri* 2457T (0.39 µM) over the course of the experiment. (**B**) The pharmacodynamics of Tebipenem in piglets over 6 hr after the administration of 50 mg/kg oral Tebipenem pivoxil in *Shigella* infected (blue) (n = 4) and uninfected (red) (n = 2) animals. The lower limit of quantitation (LLOQ) and the estimated MIC are shown. (**C**) Plots showing the concentration (ng/ml) of Tebipenem in the blood (black), duodenum (blue), and jejunum (red), of mice up to 240 min after oral administration of 50 mg/kg Tebipenem pivoxil. (**D**) Plots showing the concentration (ng/ml) of Tebipenem in the blood (black), ileum (green), caecum (purple), and colon (orange) of mice up to 240 min after oral administration of 50 mg/kg Tebipenem pivoxil. (**E**) MALDI imaging data showing the localisation of active Tebipenem in different sections of the gastrointestinal tract (duodenum to colon) of mice after oral administration of Tebipenem and Tebipenem pivoxil 15 min (left) and 60 min (right) after administration. Drug intensity scale (0–60%) and size scale are shown. A histology section of each compartment of the gastrointestinal tract is shown to highlight the localisation of active drug in the tissue or in the lumen.

We assessed the pharmacokinetics in mice of intravenous, subcutaneous, and oral doses of Tebipenem and oral doses of Tebipenem pivoxil (the oral ester prodrug of Tebipenem). Groups of mice (n = 3 in each group) were administered either 5 mg/kg of Tebipenem intravenously, 39 mg/kg of Tebipenem subcutaneously, 39 mg/kg of Tebipenem orally, or 50 mg/kg of Tebipenem pivoxil orally (equivalent amount of Tebipenem free base). The serum concentration of Tebipenem peaked in all routes of administration within 1 hr and then declined, with 39 mg/kg of oral Tebipenem showing the lowest concentration in the blood at all time points (up to 6 hr) (Figure 4A). However, the systemic concentration of active drug was equivalent between the 39 mg/kg of Tebipenem subcutaneously dosing and the 50 mg/kg of Tebipenem pivoxil given orally over the course of the 6-hr experiment (Figure 4A). Notably, the blood concentrations of subcutaneous Tebipenem and oral Tebipenem pivoxil were comparable, with peak concentration (Cmax) values of 71 and 62 µg/mL and systemic exposure area under the curve (AUC)_0-t_ of 80 and 94 µg·hr/mL, respectively (Table 2). Overall, we found that the oral bioavailability of Tebipenem when dosed as Tebipenem pivoxil pro-drug was ~52%, approximately 10-fold greater in mice than oral Tebipenem as a free base (*F*~5%); 39 mg/kg. Tebipenem free base administered orally had analogous bioavailability after 3 hr as 5 mg/kg Tebipenem free base given intravenously (Figure 4A and Table 2).

**Table 2.** The pharmacokinetics of Tebipenem and Tebipenem pivoxil.

Value	Mice			Piglets	Paediatric population (Sato et al., 2008)
	Tebipenem SQ (n = 3)	Tebipenem PO(n = 3)	Tebipenem-Pivoxil PO(n = 3)	Tebipenem-Pivoxil PO(n = 2)	Tebipenem-Pivoxil PO
C_max_ (µg/ml)	71.0 ± 15.3	3.7 ± 0.9	61.6 ± 3.9	9.0 ± 0.8	7.5 ± 3.9
T_max_ [Range] (hr)	[0.25 0.25]	[0.5 1]	[0.25 0.5]	[2 2]	0.7 ± 0.2
AUC_0-tlast_ (hr*µg/mL)	80.4 ± 7.7	7.5 ± 1.5	93.8 ± 2.7	32.6 ± 3.9	16.1 ± 3.3
Bioavailability (%)	44 ± 4	4 ± 1	52 ± 1	ND	ND

We additionally measured the Cmax and AUC of Tebipenem in gnotobiotic piglets. Two hours after an oral administration of 50 mg/kg of Tebipenem the Cmax reached a mean of 8 µg/ml in the piglets (Figure 4B and Table 2), which was equivalent to 7.5 µg/ml reported in a paediatric population (Sato et al., 2008). Longitudinally (over 6 hr), the concentration of Tebipenem declined to 1 µg/ml in the blood (Figure 4B). The AUC_0-last_ determined in piglets was higher than AUC_0-last_ reported in paediatric populations (32 vs 16 hr*µg/ml, Table 2) but was within a comparable range (Sato et al., 2008).

Oral administration of Tebipenem pivoxil may theoretically facilitate release of the active ingredient (Tebipenem) at the site of enteric infections. Aiming to test this hypothesis, we conducted quantitative LC-MS and qualitative MALDI-TOF analysis to assess the distribution of Tebipenem pivoxil and Tebipenem free base within different sections of the intestinal tract of mice after oral administration of Tebipenem pivoxil. We quantified the concentration of Tebipenem and Tebipenem pivoxil by LC-MS at 15, 30, 60, and 240 min after oral administration of 50 mg/kg Tebipenem pivoxil in the different sections of the intestinal tract (the prodrug hydrolyses ex vivo; therefore, it was not possible to differentiate between pivoxil and free base). We observed high concentrations ( > 300 µg/g tissue) of Tebipenem in the duodenum and jejunum 15 min after administration, which declined to >400 ng/g tissue after 240 min (Figure 4C). Peak drug concentrations were reached in the ileum (15 µg/g tissue) after 30 min and in the caecum (11 µg/g tissue) and colon (6 µg/g tissue) after 60 min. The highest concentrations of the drug after 4 hr were found in the caecum (Figure 4D).

We next conducted MALDI imaging of the different intestine sections at 15- and 60 min post administration, aiming to estimate the distribution of Tebipenem pivoxil and the hydrolysed free base upon oral administration of the pro-drug. The imaging from the early time point indicated that Tebipenem pivoxil could be detected in the intestinal lumen in the jejunum and the ileum, but not in the caecum and the colon (Figure 4E). At the same early time point, Tebipenem free base could be detected both in the lumen and associated with enterocytes in the jejunum and the ileum. Notably, the free base appeared to be concentrated in cells lining the caecum after 15 min. At the later time point (60 min), Tebipenem free base could be detected in the lumen and enterocytes throughout the gut, whereas Tebipenem pivoxil was not detectable in the lumen (Figure 4E). These data, in combination the oral pharmacokinetics data, showing Tebipenem pivoxil has 50% (%*F* = 52 ± 1, Table 2) bioavailability, suggest that ~50% of Tebipenem pivoxil is hydrolysed in the intestinal lumen, where the free drug may inhibit growth of bacteria present in the lumen. Additionally, 50% of the dosed Tebipenem pivoxil can penetrate the enterocytes lining the gut surface and is immediately hydrolysed (Tebipenem pivoxil pro-drug was not detected within enterocytes by MALDI-TOF), making the drug ideal for the treatment of invasive gut bacteria. Overall, MALDI imaging after 60 min indicated lower concentrations of Tebipenem free base in the lumen of jejunum, ileum, caecum, and colon, which was compatible with observations from the quantitative LC-MS analysis (Figure 4E).

**Figure 5.**
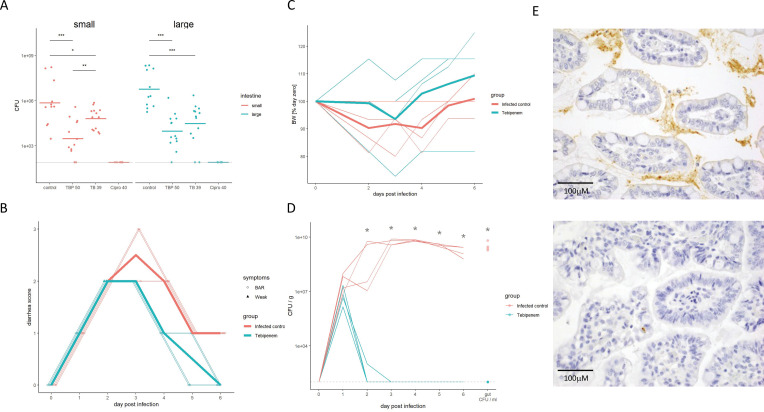
The efficacy of Tebipenem against *Shigella* in animal infections models. (**A**) Measuring the efficacy of 50 mg/kg oral Tebipenem pivoxil and 39 mg/kg oral Tebipenem by measuring CFU of *S*. *flexneri* 2457T in the small intestine (left) and large intestine (right) 24 hr after infection with a x10^7^ intraperitoneal dose of *S. flexneri* 2457T in comparison to no drug and 40 mg/kg ciprofloxacin (10 animals per group). Data shown represent mean ± the standard deviation and were determined to be normally distributed and analysed using t-tests. (**B**) Plot showing the diarrhoea score of treated (green) and untreated (red) piglets over the 6-day infection. Individual animal profiles are shown by the individual fine lines with heavy lines used to show the corresponding daily group means. The two groups differ statistically (p < 0.001) by AUC. (**C**) Plot showing the change in weight of the treated (green) and untreated (red) piglets over the six-day infection. The thin lines show individual pigs whilst the thick lines the corresponding averages. There was no significant different in weight loss between the two groups over the course of the experiment. (**D**) The efficacy of 50 mg/kg oral Tebipenem pivoxil (n = 4 animals, green) given daily for 5 days in reducing the CFU/g of faeces (every 24 hr) after infection with a 1 × 10^9^ oral dose of *S. flexneri* 2457T compared to no drug (n=4 animals, black) over 6 days. Data shown represents mean ± standard deviation. (**E**) Sections of the large intestine taken on day six post infection in a gnotobiotic piglet treated with (top) or without (lower) 50 mg/kg oral Tebipenem pivoxil after infection with *S. flexneri* 2457T observed by Immunohistochemical (IHC) staining (scale shown).

To determine if an increased systemic concentration of active drug by orally dosing the pivoxil prodrug resulted in superior performance, we compared the efficacy of Tebipenem pivoxil and Tebipenem free base in a mouse model of shigellosis. Twenty-four hours after an intraperitoneal infection with 1 × 10^7^ *S. flexneri* 2457T*,* the administration of 50 mg/kg of oral Tebipenem pivoxil resulted in a 2.3-log reduction of organisms in the small intestine compared to no antimicrobial (Figure 5A). The quantity of *S. flexneri* 2457T in the small intestine in mice treated with oral Tebipenem pivoxil was significantly lower at the same time point in mice treated with 39 mg/kg of Tebipenem freebase subcutaneously (1.2-log reduction, p < 0.05; independent t-test) (Figure 5A). We observed a comparable trend with respect to a reduction in *Shigella* CFUs in the large intestine (p < 0.05; independent t-test). Notably, neither formulation reduced the bacterial load in the small or large intestine to below detectable limits and did not perform as well as 40 mg/kg of oral ciprofloxacin.

Aiming to exploit a more physiological infection model of *Shigella* we measured the efficacy of Tebipenem pivoxil in gnotobiotic piglets using an oral 50 mg/kg dose of Tebipenem pivoxil (equivalent to the licenced 6 mg/kg dose in Japan). We infected 10 gnotobiotic piglets orally with 1 × 10^9^ CFU/ml *S*. *flexneri* 2457T; two control animals were uninfected and treated with Tebipenem pivoxil. Treatment was started 24 hr after infection; four animals were treated with oral Tebipenem pivoxil (50 mg/kg), two animals were treated with oral ciprofloxacin (30 mg/kg), and four received the formulation vehicle (1% methyl cellulose). Tebipenem pivoxil significantly reduced the severity of diarrhoea and frequency of diarrheal stools in infected gnotobiotic piglets compared to control animals (p < 0.001 for AUC of score plots; Figure 5B). Over the course of the 6-day experiment, Tebipenem-treated piglet generally gained more weight than control vehicle-treated piglets, but this difference was not statistically significant (Figure 5C). A single dose of Tebipenem pivoxil given 24 hr after infection reduced the CFU/g *Shigella* in stool from 1 × 10^7^ to zero within 24 hr, whilst untreated animals maintained a high concentration of *Shigella* in their stool for the duration of the experiment (Figure 5D). Necropsy and histopathology of the large intestine in Tebipenem pivoxil- and placebo-treated piglets was performed to compare intestinal lesions, inflammation, and the presence of intracellular *Shigella*. The histopathology showed a marked reduction in intestinal lesions and inflammation between the placebo-treated group and the group who received Tebipenem pivoxil; with the sections showing an obvious reduction in both inflammatory cells and infecting organisms (Figure 5E).

## Discussion

AMR poses one of the greatest current challenges in global health; therefore, new treatments and approaches are urgently needed to treat infections caused by antimicrobial resistant organisms. Here, we exploited a broad phenotypic screening strategy to identify compounds that may have utility against highly antimicrobial resistant enteric pathogens, specifically MDR/XDR *Shigella*. Ultimately, we identified and progressed Tebipenem pivoxil (a licenced oral carbapenem) to pre-clinical testing for *Shigella* infections. Tebipenem pivoxil has many suitable characteristics for the treatment of infections caused by MDR and XDR Gram-negative bacteria; it is broad spectrum, it is rapidly bactericidal, it is already used in paediatric clinical practice (Kataoka et al., 2016), and it can be administered orally (Arends et al., 2019), making it highly suited for the treatment of gastrointestinal infections in the community. Despite these positives, there are potential issues surrounding the widespread use of this drug, most notably the potential to generate resistance against carbapenems, a very important last resort class of antibacterial compounds.

We initiated the project by screening a large compound library against a single *Shigella* isolate grown extracellularly. Notably, none of the chemicals meeting the desired criteria (inhibition of bacterial growth with a physicochemical and safety profile suitable for downstream developability) represented novel chemical classes. All hits were already known to possess antibacterial properties and belonged to either the GSK antibacterial subset or were already commercially available. The fact that no new modes of action were identified after screening 1.7 million compounds via extracellular in vitro screening is concerning, suggesting we may be close to exhausting the chemical space for antibacterial agents that can be identified by traditional antibacterial screening assays. However, innovative phenotypic assays, which interrogate other stages of the bacterial infection lifecycle, such as intracellular activity or invasion blockade, may offer new opportunities for developing new chemical diversity, novel modes of action and possibly narrower spectrum drugs. Using the selected approach, we opted to focus on a defined set of compounds with known antibacterial activity against *E. coli*, allowing us to identify two main chemical classes that additionally exhibited activity against clinical *Shigella* isolates. Specifically, we found that carbapenems had high potency against both laboratory *Shigella* strains and clinical isolates of MDR *Shigella* from highly endemic LMICs (Thanh Duy et al., 2020). Consequently, Tebipenem was selected as a lead candidate for further pre-clinical characterisation due to the great potency and the availability of a prodrug which allows oral administration.

Tebipenem had enhanced activity (with respect to ciprofloxacin) against a panel of 81 *Shigella* clinical isolates from Southeast Asia, an observation in line with the high prevalence of ciprofloxacin resistance in many *Shigella sonnei* and a high proportion of Gram-negative bacterial pathogens across Southeast Asia (Chung The et al., 2016; Pham Thanh et al., 2016). Tebipenem additionally exhibited high level of potency against a broad panel of >300 clinically relevant MDR enteric pathogens. These data outline the potential use of this antimicrobial for the treatment of enteric infections of suspected bacterial origin without the necessity of an accompanying diagnostic test, which remains the most common clinical practice in many LMICs. It is noteworthy that carbapenem resistance is emerging in some Gram-negative organisms in Asia and has spread to other regions (Yao et al., 2018; Wyres et al., 2020). This phenomenon is partly associated with the spread of the NDM-1 gene, which encodes a beta-metalloprotease (Grundmann et al., 2017; Yong et al., 2009). Carbapenem resistance has not yet been reported in any *Shigella* species and is not currently common in other Gram-negative bacteria causing diarrhoea. However, given the propensity of *Shigella* to develop resistance to commonly used antimicrobials through the acquisition of AMR plasmids and individual gene cassettes, we additionally would need to consider the requirement of a companion diagnostic to avoid treatment failures or overemployment of the drug, which may trigger resistance. Further, we need a broader discussion on the operational and ethics of using oral carbapenems and their access. If this is a route that is to be followed we clearly need patient efficacy data, antimicrobial clearance data, projection modelling, and detailed insights into collateral effects, including the selection and generation of carbapenem resistant organisms. We see the need for some legislation for the use of Tebipenem in this context, but also recognise that we have limited alternatives as we have depleted the pool of efficacious antimicrobials.

We considered that the Tebipenem (and other carbapenems) may be synergistic with drugs with alternative other modes of action, including existing standard of care antimicrobials (Williams and Berkley, 2018). Antibacterial combinations are an understudied area, and we did not aim measure the likelihood of emergence of resistance to either compound, just to identify potential synergy/inhibition. These preliminary experimental data suggested that Azithromycin, which reduced the MIC for *Shigella* isolates for Tebipenem when tested in in vitro combination, may offer an encouraging primary combination option, given the common use of azithromycin for the treatment of severe diarrhoea (Gharpure et al., 2022). Additionally, a second potential combination showing in vitro synergy was identified with an LpxC inhibitor (Surivet et al., 2020), which again showed good activity when tested against the majority of enteric bacteria studied. Again, this observation is encouraging and adds further support to LpxC development programs; however, there is a clear need to better study antibacterial combinations and not only to study synergy but also if they do have an impact on stimulating resistance to newer classes of antimicrobials (Zhu et al., 2021). Our data highlight the utility of Tebipenem for treating a spectrum of enteric pathogens and the potential benefit of co-administration with recommended antimicrobials suggest that combination therapy with Tebipenem is clearly worth exploring through clinical trials and/or through controlled human infection studies with *Shigella* (MacLennan et al., 2019). The existing safety profile of tebipenem and other standard-of-care antimicrobial opens the possibility of direct progression to a clinical proof of concept study as soon as possible (Kataoka et al., 2016).

Encouragingly, Tebipenem pivoxil is approved and marketed in Japan as Orapenem (Meiji Seika) for the treatment of adult and paediatric respiratory tract infections (Kataoka et al., 2016). In addition, Spero Therapeutics are working on a novel formulation with extended half-life for administration in adults which is under clinical evaluation for recurrent urinary tract infections (Arends et al., 2019; Eckburg et al., 2019). Notably, the MICs of the panel of enteric bacteria against Tebipenem tested here were substantially lower than the clinical breakpoints reported for efficacious treatment of respiratory tract infections. *Shigella* infections have a pathophysiology which is obviously distinct to that of respiratory tract infections, but our MALDI-TOF data suggests that Tebipenem reaches a high concentration in the enterocytes lining the gastrointestinal surface. Our data suggest that the currently approved dose of Orapenem will reach higher local concentrations in the *Shigella* infection-relevant cells in the gut than in the respiratory tract. Therefore, the drug distribution behaviour of Orapenem appears to be especially convenient for the treatment of enteric infections, as the active component is specifically released at the site of infection. The release of free Tebipenem intracellularly would clear intracellular bacteria (vital for *Shigella* and *Salmonella*) and active Tebipenem in the intestinal lumen would reduce extracellular bacteria and limit cellular invasion. Our data from two distinct animal models implies this process is occurring, as the drug was highly potent after *Shigella* challenge and able to clear the gut of infecting organisms with 24 hr of administration in gnotobiotic piglets.

In conclusion, we performed a screening study with the specific aim of identifying novel compounds with antibacterial activity against MDR/XDR *Shigella*. Overall, the results were disappointing and raise wider questions about the potential for the discovery of new antibacterial classes. However, we did identify multiple new avenues for using existing compounds; specifically, Tebipenem had excellent in vitro profile against MDR/XDR *Shigella* and other Gram-negative bacteria (Mylona et al., 2021). We could show that the oral bioavailability of and pharmacokinetics of Tebipenem pivoxil makes it ideal for the treatment of gastrointestinal infections. Experimentation in animal models of shigellosis found the drug to be highly efficacious with comparable activity to ciprofloxacin when animals were challenged with ciprofloxacin susceptible *Shigella* isolates.

We are in desperate need of alternative antimicrobials for infections caused by MDR/XDR bacteria and our data outline the oral carbapenem Tebipenem as an ideal repurposing candidate. The testing and controlled introduction of Tebipenem pivoxil combinations for MDR *Shigella* infections may slow the rate of spread of organisms that exhibit resistance to current standard of care antimicrobials or provide an extended window of opportunity for the further development and introduction of *Shigella* vaccines and the next generation of therapeutic approaches.

## Data Availability

All raw data for this project is available at https://doi.org/10.5281/zenodo.5929105. Exceptions include the propriety compound list owned by GSK. Access to compounds can be requested via the open lab foundation and GSK and the raw MALDI-TOF data are available upon request from the corresponding authors. The following dataset was generated: BakerS 2021The repurposing of tebipenem pivoxil as alternative therapy for severe gastrointestinal infections caused by extensively drug resistant Shigella sppZenodo10.5281/zenodo.4783491PMC895960035289746

## Additional files

Supplementary file 1. Antimicrobial susceptibility profile of experimental organisms.

Supplementary file 2. Profile and MIC ranges of marketed penem antimicrobials evaluated against *S. flexneri* EG478 and *S. sonnei* 02–1181.

Supplementary file 3. Rates of spontaneous in vitro resistance for *S. flexneri* 2457T against a range of antimicrobials.

Transparent reporting form
