# Correction: The repurposing of Tebipenem pivoxil as alternative therapy for severe gastrointestinal infections caused by extensively drug-resistant *Shigella* spp

**DOI:** 10.7554/eLife.83117

**Published:** 2022-09-05

**Authors:** Elena Fernández Álvaro, Phat Voong Vinh, Cristina de Cozar, David R Willé, Beatriz Urones, Alvaro Cortés, Alan Price, Nhu Tran Do Hoang, Tuyen Ha Thanh, Molly McCloskey, Shareef Shaheen, Denise Dayao, Amanda Martinot, Jaime de Mercado, Pablo Castañeda, Adolfo García-Perez, Benson Singa, Patricia Pavlinac, Judd Walson, Maria Santos Martínez-Martínez, Samuel LM Arnold, Saul Tzipori, Lluis Ballell Pages, Stephen Baker

**Keywords:** Other

Álvaro EF, Vinh PV, de Cozar C, Willé DR, Urones B, Cortés A, Price A, Hoang NTD, Thanh TH, McCloskey M, Shaheen S, Dayao D, Martinot A, de Mercado J, Castañeda P, García-Perez A, Singa B, Pavlinac P, Walson J, Martínez-Martínez MS, Arnold SLM, Tzipori S, Pages LB, Baker S. 2022. alternative therapy for severe gastrointestinal infections caused by extensively drug-resistant *Shigella spp*. *eLife* **11**:e69798. doi: 10.7554/eLife.69798.

The final version of this paper included histopathology analysis of all the animals involved in the evaluation of the therapeutic efficacy of tebipenem pivoxil in gnotobiotic piglets. Amanda Martinot was a key contributor to this piece as she read the histopathology of all the experimental animals, provided interpretation of the histology results, and provided the micrographs. Therefore, we request the inclusion of Amanda Martinot in the author’s list.

## New authors list:

Elena Fernández Álvaro, Phat Voong Vinh, Cristina de Cozar, David R Willé, Beatriz Urones, Alvaro Cortés, Alan Price, Nhu Tran Do Hoang, Tuyen Ha Thanh, Molly McCloskey, Shareef Shaheen, Denise Dayao, Amanda Martinot, Jaime de Mercado, Pablo Castañeda, Adolfo García-Perez, Benson Singa, Patricia Pavlinac, Judd Walson, Maria Santos Martínez-Martínez, Samuel LM Arnold, Saul Tzipori, Lluis Ballell Pages, Stephen Baker

## Original authors list:

Elena Fernández Álvaro, Phat Voong Vinh, Cristina de Cozar, David R Willé, Beatriz Urones, Alvaro Cortés, Alan Price, Nhu Tran Do Hoang, Tuyen Ha Thanh, Molly McCloskey, Shareef Shaheen, Denise Dayao, Jaime de Mercado, Pablo Castañeda, Adolfo García-Perez, Benson Singa, Patricia Pavlinac, Judd Walson, Maria Santos Martínez-Martínez, Samuel LM Arnold, Saul Tzipori, Lluis Ballell Pages, Stephen Baker

## Details for the omitted authors

### Amanda Martinot

Department of Infectious Disease and Global Health, Tufts University Cummings School of Veterinary Medicine, North Grafton, United States.

Contribution: Formal analysis, Methodology, Validation, Visualization, Histopathology

Competing interests: No competing interests declared

All authors have agreed to the change to add the omitted author to the author list.

We also incorrectly spelt Saul Tzipori’s name in the published article, where their first name had been swapped with their surname. Their name has now been corrected to read Saul Tzipori.

The article has been corrected accordingly.

