# Supplementary material for: The repurposing of Tebipenem pivoxil as alternative therapy for severe gastrointestinal infections caused by extensively drug-resistant Shigella spp
Source: eLife. 2022 Mar 15;11:e69798. doi: 10.7554/eLife.69798 (PMC8959600; doi:10.7554/eLife.69798)
Supplement: Peer review history

# Editor report: Editor's evaluation

**DOI:** 10.7554/eLife.69798.sa0

**Authors:** María Mercedes Zambrano

**Affiliations:** https://ror.org/03npzn484 CorpoGen Colombia

Through a high throughput screen, the authors identify an oral carbapenem antibiotic, Tebipenem, effective against susceptible and multi-drug resistant Shigella strains and other enteric pathogens. This antibiotic was also effective when combined with other antibiotics and thus shows promise as a repurposed alternative for the treatment for severe diarrhea.

# Decision letter

**DOI:** 10.7554/eLife.69798.sa1

**Affiliations:** https://ror.org/03npzn484 CorpoGen Colombia; https://ror.org/01nrxwf90 University of Edinburgh United Kingdom

In the interests of transparency, eLife publishes the most substantive revision requests and the accompanying author responses.

**Decision letter after peer review:**

Thank you for submitting your article "The repurposing of tebipenem pivoxil as alternative therapy for severe gastrointestinal infections caused by extensively drug resistant *Shigella* spp." for consideration by *eLife*. Your article has been reviewed by 2 peer reviewers, and the evaluation has been overseen by a Reviewing Editor and Wendy Garrett as the Senior Editor. The following individual involved in review of your submission has agreed to reveal their identity: Luke McNally (Reviewer #2).

The reviewers have discussed their reviews with one another, and the Reviewing Editor has drafted this to help you prepare a revised submission.

Essential revisions:

While the work is valuable and of broad interest, in its present form it is difficult to fully assess its impact and the validity of the claims. In particular, the authors should include information on sample sizes and statistical analyses, which are essential to support the findings. Additional information should also be included regarding the relevance of acquiring resistance to tebipenem and /or combination therapy.

1) Overall, the presentation of methods and results are incomplete and hard to follow, making it hard to judge the rigor and robustness of the underlying science. Specifically, important information about replication and statistical analysis are missing for many experiments and there are multiple cases where results and conclusions are discussed without any data being shown. This limits the utility and impact of the paper as it would be impossible for any of the experiments as presented to be used in meta-analyses. Some examples are listed below:

– For all figure panels summary statistics and results of statistical tests should be reported. The statistical tests that seem to be carried out only have p-values reported, which is not sufficient. Test statistics and degrees of freedom need to also be reported.

– Figure 3 plots "average FICI score" but the number of replicates used to generate the averages are not stated, there are no error bars to show variation from the average, and no statistical tests are described.

– Lines 167-169: The authors state that the frequency of spontaneous tebipenem-resistance mutations was "in a low range compared to other antimicrobials (<1 x 10-9)." However, these data are not presented in a figure or table and there is no indication of number of replicates (especially important for stochastic processes such as mutagenesis), error values, or frequency values for other antimicrobials.

– Figure 4A and B show MALDI-TOF images of intestinal sections. These seem to be representative images (single section from single animal) but no summary data about multiple sections per animal or replicate animals are presented. The Methods state that 3 animals per group were used (line 499), so where are the data? Also, the scales in the images are impossible to read and the general phenomenon being observed is poorly described in the text. As a non-expert, I have no idea what this figure is attempting to show.

– Figure 5B-D represent experiments performed in 2-4 piglets per treatment (Methods, lines 559-562) but there are no error bars on data points (Figure 5B) and single images are shown in C and D with no reference to summary data across images/animals.

– Lines 254-255: "Tebipenem pivoxil reduced the frequency of diarrheal stools in infected piglet, performing in an equivalent fashion to the ciprofloxacin control." The underlying data are not presented anywhere.

– Only the results presented in Figure 4D appear to have had any formal statistical analysis, and the results of this test are not adequately reported.

– No detail is given on the statistical procedures for hit selection other than stating that these used a standard GSK protocol. These details should also be given in the methods also.

2) An additional concern, is that the authors claim that the synergistic combination of tebipenem and an LpxC inhibitor should reduce the emergence of resistance to treatment. However, no data is presented directly assessing if combination therapy actually impacts the rate of spontaneous emergence of resistance during treatment. Without this direct evidence it is unclear how this synergy would actually affect resistance evolution.

3) While the synergy of tebipenem and LpxC is an interesting and potentially important result, it's evolutionary implications are far from straightforward, as it is not clear that synergistic multi-drug therapy will actually slow resistance evolution (e.g. https://journals.plos.org/plosbiology/article/info:doi/10.1371/journal.pbio.1001540). This claim either needs to be backed up by comparing the rate of emergence of resistance under monotherapy and combination therapy, or these claims should be toned down and caveated throughout the manuscript.

4) The discussion of potential clinical utility of tebipenem pivoxil and drug cocktails including pivoxil is somewhat contradictory and concerning. The authors suggest that, due to its broad-spectrum activity against clinical isolates of multiple MDR enteric pathogens, tebipenem pivoxil could be used as a first-line therapy without diagnostic/drug-susceptibility profiling. I agree that this is appealing in resources-poor settings, but could lead to rampant overuse and drive rapid emergence of resistance. Carbapenems are typically reserved for otherwise intractable infections, and so increased selection for carbapenem-resistant enteric pathogens and increased circulation of mobile element-encoded carbapenemases could impact treatment of diverse MDR pathogens and pose a serious public health threat. To combat resistance, the authors suggest use of combination therapy. However, they discuss azithromycin as one of the most promising candidates for combination therapy, despite its apparent antagonistic interaction with tebipenem against *E. coli*. This suggests that combination therapy should only be used after diagnostic testing, limiting the clinical utility of this approach in many settings. My conclusion is that tebipenem pivoxil could be a valuable tool in certain circumstances, but it is unclear how to responsibly use it at scale, which limits the potential impact on global health.

5) More discussion is about the authors proposal to use tebipenem as a treatment for diarrhoea disease in LMICs regarding the potential cross resistance to other carbapenems. Carbapenems are often used as last-line drugs for seriously ill hospitalised patients. If resistance to tebipenem confers cross resistance to other carbapenems the public health implications of widespread tepipenem use could be highly complex. This is a serious public health and ethical question surrounding the use of tebipenem as proposed. I think some added discussion of what needs to be assessed/quantified to weigh up this risk would really strengthen the manuscript. For example, we need to quantify bystander selection on other gut microbes from tebipenem usage, and assess the degree of cross-resistance of tebipenem resistant mutants to other carbapenems.

# Reply: Author response

**DOI:** 10.7554/eLife.69798.sa2

Essential revisions:

While the work is valuable and of broad interest, in its present form it is difficult to fully assess its impact and the validity of the claims. In particular, the authors should include information on sample sizes and statistical analyses, which are essential to support the findings.

Information about sample sizes as well as statistical analyses in all experiments has been included.

Additional information should also be included regarding the relevance of acquiring resistance to tebipenem and /or combination therapy.

Detailed information about in vitro evaluation against clinical isolates, spontaneous resistant mutant generation and combinations has been included. We have also considered the very useful reviewer´s comments in this respect and generated a more comprehensive discussion that takes in consideration the potential benefits and risks.

1) Overall, the presentation of methods and results are incomplete and hard to follow, making it hard to judge the rigor and robustness of the underlying science. Specifically, important information about replication and statistical analysis are missing for many experiments and there are multiple cases where results and conclusions are discussed without any data being shown. This limits the utility and impact of the paper as it would be impossible for any of the experiments as presented to be used in meta-analyses. Some examples are listed below:

– For all figure panels summary statistics and results of statistical tests should be reported. The statistical tests that seem to be carried out only have p-values reported, which is not sufficient. Test statistics and degrees of freedom need to also be reported.

Test statistics have been reported for all experiments performed and included in the figure legends.

– Figure 3 plots "average FICI score" but the number of replicates used to generate the averages are not stated, there are no error bars to show variation from the average, and no statistical tests are described.

Number of replicates is specified in Figure legend and error bars have been included to the Figure showing average values.

– Lines 167-169: The authors state that the frequency of spontaneous tebipenem-resistance mutations was "in a low range compared to other antimicrobials (<1 x 10-9)." However, these data are not presented in a figure or table and there is no indication of number of replicates (especially important for stochastic processes such as mutagenesis), error values, or frequency values for other antimicrobials.

Additional information about the FoR experiment has been added. This includes a table with the frequency of resistance data obtained for other antibiotics as well as the number of replicates.

– Figure 4A and B show MALDI-TOF images of intestinal sections. These seem to be representative images (single section from single animal) but no summary data about multiple sections per animal or replicate animals are presented. The Methods state that 3 animals per group were used (line 499), so where are the data? Also, the scales in the images are impossible to read and the general phenomenon being observed is poorly described in the text. As a non-expert, I have no idea what this figure is attempting to show.

The MALDI-TOF section, that is part of an experiment to assess pro-drug hydrolysis and drug distribution along the intestine of mice, has been completely rewritten in the experimental and Results sections of the manuscript. We agree with the reviewers that the previous version was very difficult to follow and have tried to generate a new comprehensive version.

– Figure 5B-D represent experiments performed in 2-4 piglets per treatment (Methods, lines 559-562) but there are no error bars on data points (Figure 5B) and single images are shown in C and D with no reference to summary data across images/animals.

We have redone the piglet efficacy Figure and include data from each individual animal as well as information about statistical analysis and significance. We have uploaded all raw images to the file share site.

– Lines 254-255: "Tebipenem pivoxil reduced the frequency of diarrheal stools in infected piglet, performing in an equivalent fashion to the ciprofloxacin control." The underlying data are not presented anywhere.

This has been added to Figure 5

– Only the results presented in Figure 4D appear to have had any formal statistical analysis, and the results of this test are not adequately reported.

We have included information about statistical analysis and statistical significance in all experiments and figures.

– No detail is given on the statistical procedures for hit selection other than stating that these used a standard GSK protocol. These details should also be given in the methods also.

Following the very pertinent reviewer´s comments, we have included a detailed information of the high throughput screening process and hit selection, completing both the results and experimental section. We hope that this new version provides clearer, easy to follow information.

2) An additional concern, is that the authors claim that the synergistic combination of tebipenem and an LpxC inhibitor should reduce the emergence of resistance to treatment. However, no data is presented directly assessing if combination therapy actually impacts the rate of spontaneous emergence of resistance during treatment. Without this direct evidence it is unclear how this synergy would actually affect resistance evolution.

We have considered this comment and modified our manuscript discussion specifying that we tested for synergism rather than for frequency of resistance comparing single-compounds and combinations.

3) While the synergy of tebipenem and LpxC is an interesting and potentially important result, it's evolutionary implications are far from straightforward, as it is not clear that synergistic multi-drug therapy will actually slow resistance evolution (e.g. https://journals.plos.org/plosbiology/article/info:doi/10.1371/journal.pbio.1001540). This claim either needs to be backed up by comparing the rate of emergence of resistance under monotherapy and combination therapy, or these claims should be toned down and caveated throughout the manuscript.

We have clarified the aim and results of the experiments performed and toned down our claims.

4) The discussion of potential clinical utility of tebipenem pivoxil and drug cocktails including pivoxil is somewhat contradictory and concerning. The authors suggest that, due to its broad-spectrum activity against clinical isolates of multiple MDR enteric pathogens, tebipenem pivoxil could be used as a first-line therapy without diagnostic/drug-susceptibility profiling. I agree that this is appealing in resources-poor settings, but could lead to rampant overuse and drive rapid emergence of resistance. Carbapenems are typically reserved for otherwise intractable infections, and so increased selection for carbapenem-resistant enteric pathogens and increased circulation of mobile element-encoded carbapenemases could impact treatment of diverse MDR pathogens and pose a serious public health threat. To combat resistance, the authors suggest use of combination therapy. However, they discuss azithromycin as one of the most promising candidates for combination therapy, despite its apparent antagonistic interaction with tebipenem against *E. coli*. This suggests that combination therapy should only be used after diagnostic testing, limiting the clinical utility of this approach in many settings. My conclusion is that tebipenem pivoxil could be a valuable tool in certain circumstances, but it is unclear how to responsibly use it at scale, which limits the potential impact on global health.

We fully agree with this comment and have specifically included part of this feedback in the discussion. It is clear that further development and use of diagnostics test will improve the efficacy of antibiotic treatment and will prevent further resistance generation. We also agree that the specific use framework of tebipenem pivoxil needs to be further discussed, but the potential of use at least as an oral alternative to meropenem, reducing treatment costs enabling outpatient treatment, is clear to us.

5) More discussion is about the authors proposal to use tebipenem as a treatment for diarrhoea disease in LMICs regarding the potential cross resistance to other carbapenems. Carbapenems are often used as last-line drugs for seriously ill hospitalised patients. If resistance to tebipenem confers cross resistance to other carbapenems the public health implications of widespread tepipenem use could be highly complex. This is a serious public health and ethical question surrounding the use of tebipenem as proposed. I think some added discussion of what needs to be assessed/quantified to weigh up this risk would really strengthen the manuscript. For example, we need to quantify bystander selection on other gut microbes from tebipenem usage, and assess the degree of cross-resistance of tebipenem resistant mutants to other carbapenems.

This has been further discussed in the discussion
